# Gaps in Clinical Prevention and Treatment for Alcohol Use Disorders: Costs, Consequences, and Strategies

**DOI:** 10.35946/arcr.v35.2.14

**Published:** 2014

**Authors:** Mark L. Willenbring

**Affiliations:** **Mark L. Willenbring, M.D.**, *former director of Treatment and Recovery Research at the National Institute on Alcohol Abuse and Alcoholism, is founder and CEO of Alltyr: Transforming Treatment for Addictions, St. Paul, Minnesota.*

**Keywords:** Alcohol use, abuse, and dependence, heavy drinking, alcohol use disorders (AUDs), alcohol-related problems, alcohol burden, burden of disease, morbidity, mortality, prevention, treatment, prevention strategy, treatment strategy, screening and brief intervention, primary care, cost-effectiveness of AOD health services

## Abstract

Heavy drinking causes significant morbidity, premature mortality, and other social and economic burdens on society, prompting numerous prevention and treatment efforts to avoid or ameliorate the prevalence of heavy drinking and its consequences. However, the impact on public health of current selective (i.e., clinical) prevention and treatment strategies is unclear. Screening and brief counseling for at-risk drinkers in ambulatory primary care has the strongest evidence for efficacy, and some evidence indicates this approach is cost-effective and reduces excess morbidity and dysfunction. Widespread implementation of screening and brief counseling of nondependent heavy drinkers outside of the medical context has the potential to have a large public health impact. For people with functional dependence, no appropriate treatment and prevention approaches currently exist, although such strategies might be able to prevent or reduce the morbidity and other harmful consequences associated with the condition before its eventual natural resolution. For people with alcohol use disorders, particularly severe and recurrent dependence, treatment studies have shown improvement in the short term. However, there is no compelling evidence that treatment of alcohol use disorders has resulted in reductions in overall disease burden. More research is needed on ways to address functional alcohol dependence as well as severe and recurrent alcohol dependence.

Heavy drinking takes a high toll on society. Other articles in this issue summarize the disease burden and economic cost to society attributable to alcohol use, which provide a powerful incentive to develop and implement ways to reduce them. The focus of this article is on the role of selective (i.e., clinical) prevention and treatment approaches for heavy drinkers and people with alcohol use disorders (AUDs) in reducing the burden associated with excessive alcohol use. As used here, selective, or clinical, prevention refers to strategies targeted at individuals at higher risk of experiencing adverse alcohol effects, such as screening and brief counseling of heavy drinkers in health care settings or internet-based screening and advice provided to college students. The term “treatment” refers to services for alcohol dependence provided by a professional, such as a counselor, social worker, nurse, psychologist, or physician. Community peer-led support groups such as Alcoholics Anonymous are considered to be distinct from professional treatment services, much like a diabetes support group would be distinguished from endocrinology services. The article focuses on the following three questions: (1) Can selective prevention and treatment reduce the disease burden attributable to heavy drinking? (2) Are some treatment approaches more cost-effective than others? (3) Do gaps exist in the current continuum of care? After addressing these issues, the review suggests research priorities to help close existing gaps and reduce the burden of disease.

## Selective Prevention and Treatment: Effectiveness, Cost-Effectiveness, and Disease Burden

Screening and brief advice for at-risk (i.e., nondependent) drinkers, commonly known as screening and brief intervention (SBI), is effective at reducing drinking for a year or more and in many studies also has been shown to reduce alcohol-related harms, such as motor-vehicle crashes and driving violations. Its efficacy is supported by numerous randomized controlled trials and multiple meta-analyses; as a result, the U.S. Prevention Task Force has listed it as a Type B recommendation for medical prevention services (Babor et al. 2007; Whitlock et al. 2004). The evidence is strongest for nondependent heavy drinkers who present for primary care services in ambulatory settings. Unfortunately, a recent meta-analysis of studies of SBI in primary care settings failed to show significant reductions in subsequent health care utilization (Bray et al. 2011). The efficacy of SBI in other settings, such as emergency departments (EDs) or hospitals, has not been established, although several randomized controlled trials have been conducted (Field et al. 2010). One explanation for the observed differences may be the patient populations analyzed. Thus, in most of the outpatient primary care studies, participants with alcohol dependence were excluded from the analysis, whereas that generally was not the case for studies conducted in EDs or hospital settings. Moreover, patients with alcohol dependence are much more commonly encountered in ED and hospital settings than in primary ambulatory care. In summary, at this time, SBI in primary care ambulatory settings for adults can be strongly recommended as highly efficacious, whereas SBI in EDs or hospitals cannot.

SBI also seems to be effective among select groups when delivered through internet-based or computerized applications. In particular, there is strong evidence that digital SBI can effectively reduce drinking and associated consequences among college students (Moreira et al. 2009). It is not clear whether or to what extent this finding might generalize to other population subgroups, but it is certainly plausible that it could, provided the target population has easy access to computers and is computer literate. The same holds true for other methods, such as telephone-based SBI or use of the relatively new publication and Web site called Rethinking Drinking, which is published by the National Institute on Alcohol Abuse and Alcoholism (NIAAA).

Despite the evidence supporting its effectiveness, SBI is not yet being implemented widely (Hingson et al. 2012). Widespread dissemination of information about recommended drinking limits and easy access to screening and brief counseling has the potential to make a significant public health impact. Because at-risk drinkers are much more numerous than alcohol-dependent people, at-risk drinking contributes a much greater disease burden than alcohol dependence. Accordingly, widespread implementation of SBI has the potential to reduce a greater proportion of disease burden than even very effective treatment, a concept known as the prevention paradox (Rose 1981). Therefore, more research is needed to expand the implementation of SBI in the at-risk population and further increase its effectiveness.

Estimating the effectiveness and cost-effectiveness of treatment is more complex. Most reviews conclude that treatment is effective at reducing drinking and associated consequences. Multiple behavioral treatment approaches—such as cognitive– behavioral therapy, motivational enhancement therapy, 12-step facilitation, behavioral marital therapy, and community reinforcement—have similar and relatively high levels of short-term success in reducing drinking and associated consequences, at least when treatment is provided by the highly trained, motivated, and closely supervised clinicians participating in clinical efficacy trials (Project MATCH Research Group 1998). Why these technically diverse counseling techniques produce almost identical drinking outcomes is unclear. Three alternative explanations have been offered:

- The specific technique is less important than other, mostly unidentified, factors associated with psychotherapy.
- Each approach works via different mechanisms but produces similar results on average, much like different antidepressants acting through different mechanisms produce similar outcomes in the treatment of depression.
- Professional treatment only has a small effect in determining outcome compared with other, nontreatment factors, such as social control (e.g., driving-while-intoxicated laws, family pressure, or employer mandate), natural history of alcohol dependence, and the tendency to revert to usual levels of drinking following resolution of a crisis where drinking had peaked (i.e., regression to the mean).

This last explanation is supported by recent research demonstrating that changes in drinking habits begin weeks before treatment entry (Penberthy et al. 2007). Likewise, in another study of treatment of alcohol dependence that examined events leading to treatment seeking (Orford et al. 2006), the findings suggested that the change point occurred prior to treatment entry. Thus, it is unclear how much of the positive change can be attributed to the treatment processes themselves as opposed to other factors leading to and following treatment seeking.

What is clear, however, is that researchers and clinicians do not yet understand how or why some people change in response to treatment and others do not. To address this issue, NIAAA led the way at the National Institutes of Health (NIH) in shifting the focus of behavioral treatment research to identifying the mechanisms of behavior change rather than encouraging more comparisons of different psychotherapy approaches (Willenbring 2007). The NIH subsequently developed a major initiative on basic behavioral research (Li 2009). This research initiative provides an opportunity to investigate many obvious questions. For example, what are the social forces that either support or impede positive health behavior change? What determines their impact, in terms of the response of the individual? Why and how do people begin to change, and what determines the resilience of that change? What is the basic science underlying behavior change, at all levels from genetic and genomic to cellular, organic, individual, and social interactions? Research elucidating the basic science of behavior change is an exciting and promising area that has the potential to substantially change the types of interventions that are available, making them more powerful, available, and cost-effective.

The lack of clarity about what causes change in drinking behavior also results in uncertainty as to whether treatment of alcohol dependence reduces disease burden. The community prevalence of alcohol dependence, which is about 4 percent in any year, has not changed substantially in recent years (Substance Abuse and Mental Health Services Administration 2011). Earlier studies found a cost offset of treatment—that is, lower health care costs after treatment than before treatment (Holder 1998). More recent studies, however, have found that heavy drinkers who are not in crisis underutilize health care, at least in an employed population, suggesting that the observed cost reduction is more a reflection of the natural history of drinking behavior and of a regression to the mean (Finney 2008; Zarkin et al. 2004). In other words, people suffering from any disease tend to seek treatment when their condition is most severe. In the case of alcohol dependence, treatment seeking therefore would be preceded by an escalation of drinking, complications, and utilization of medical services and, consequently, high costs before treatment entry. Because chronic conditions such as alcohol dependence wax and wane, most people will tend to improve after a period of greater severity, even without effective treatment, so that subsequent reduced costs may not necessarily be associated with treatment. Also, every patient’s disease trajectory is different, so that when drinkers are assessed before and after treatment, some of them will be well at followup, whereas for others their condition will be more severe. The average severity, however, will be less following treatment, because for all patients studied, their disease severity at treatment entry will have been high. The most rigorous study of cost-effectiveness of alcoholism treatment, the COMBINE trial, found that treatment was cost-effective, especially pharmacotherapy with medical management (Zarkin et al. 2008, 2010). The interpretation of these findings is limited, however, by the study’s highly rigorous trial design, intensive follow up, and exclusion criteria (Anton et al. 2006), and it is unknown to what extent these findings generalize to community treatment programs and participants.

Another limitation when estimating the effects of treatment on public health is that relatively few affected people seek treatment. For example, among people who develop alcohol dependence at some point in their lives only 12 percent seek treatment in a specialty treatment program (Hasin et al. 2007). Among people who have AUDs and who perceive a need for treatment, almost two-thirds (i.e., 65 percent) fail to obtain it because they are not ready to stop drinking or feel they can handle it on their own. Other common reasons for the failure to seek treatment include practical barriers, such as lack of health insurance, the cost of treatment, and lack of transportation or access to treatment, which are reported by 59 percent of respondents, and stigma, which is reported by 31 percent (Center for Behavioral Health Statistics and Quality 2012).^1^ Thus, more people might seek treatment if it was less expensive, stigmatizing, and disruptive than most treatment approaches. Efforts to improve access, affordability, and attractiveness of treatment, especially for individuals with less severe AUDs should be encouraged.

Despite these limitations, some tentative conclusions can be drawn as to which approaches to treating alcohol dependence are more cost effective. Studies found no significant difference in outcomes between residential and outpatient treatment and no clear relationship between intensity of treatment and outcome (Fink et al. 1985; Longabaugh et al. 1983; McCrady 1986). For example, medical management plus pharmacotherapy with naltrexone generated similar outcomes to more expensive counseling approaches, even when counseling was performed once weekly and on an outpatient basis (Anton et al. 2006; O’Malley et al. 2003). These studies suggest that a more individualized, outpatient, and medically based approach may provide a cost-effective alternative to approaches favoring intensive psycho-education, which often are provided in residential settings. Treatment provided in residential rather than outpatient settings may add considerable expense without a commensurate improvement in outcomes. In addition, confidential treatment by their usual primary care physician involving only routine clinic visits may attract more people, thus expanding access to effective treatments.

## Gaps in the Continuum of Care

There are several gaps in the continuum of care that deserve attention, affecting drinkers across the spectrum of alcohol involvement. Recent epidemiological research has demonstrated that alcohol involvement varies along a continuum ranging from asymptomatic heavy drinking (i.e., at-risk drinking), through functional alcohol dependence, and to severe and recurrent alcohol dependence (Willenbring et al. 2009). The continuum of care ideally should correspond to this epidemiology but does not at this time. Most studies and treatment approaches have focused on the more severe end of the spectrum—that is, people with severe, recurrent dependence. However, the vast majority of heavy drinkers either does not have alcohol dependence or has a relatively milder, self-limiting form (Moss et al. 2007). This spectrum of severity is similar to that for other chronic diseases, such as asthma. Likewise, examining treatment seekers in the current system of care yields similar results to studying hospitalized asthmatics: thus, heavy drinkers in treatment exhibit more severe dependence, more comorbidity, less response to treatment, and a less supportive social network compared with people who do not seek intensive treatment (Bischof et al. 2003; Dawson et al. 2005; Sobell et al. 2000). In contrast, people with functional alcohol dependence^2^ predominantly exhibit “internal” symptoms, such as impaired control; a persistent desire to cut down on their drinking but finding it hard to do; and alcohol use despite internal symptoms such as insomnia, nausea, or hangover. These individuals generally drink much less than more seriously affected people (Moss et al. 2007). Functional alcohol dependence typically resolves after a few years, mostly without requiring specialty treatment (Hasin et al. 2007). Large gaps in services exist for people at both ends of the spectrum of dependence severity—that is, both for people at the milder end of the spectrum (i.e., at-risk drinkers and people with functional alcohol dependence) and for those at the most severe end (i.e., with recurrent, treatment-refractory dependence).

There currently are few services for at-risk drinkers and people with functional alcohol dependence. In primary medical care, very few patients are screened and positive screening results addressed (McGlynn et al. 2003). Furthermore, functional alcohol dependence largely is ignored because although these individuals meet diagnostic criteria for dependence, they rarely seek treatment in the current system (Moss et al. 2007). These gaps are significant from a public health perspective because the prevalence of at-risk drinking and functional dependence is much higher than that of more severe disorders and these conditions therefore account for the majority of excess morbidity, mortality, and associated costs attributable to alcohol consumption (Centers for Disease Control and Prevention 2012). Whether wider implementation of SBI would result in a reduction in disease burden is not known at this time. However, enhancement of these approaches, especially among young people and community-dwelling heavy drinkers not seeking medical care, might reduce disease burden, although the two populations require somewhat distinct approaches. More studies of secondary prevention efforts outside of medical settings therefore are needed.

SBI in primary care settings to identify people with AUDs at the milder end of the severity spectrum is effective and may be cost-effective (Solberg et al. 2008), but many questions remain. For example, is it more cost-effective to target higher-risk groups (e.g., young people) for routine screening or is universal screening better overall? And when should screening occur (e.g., only during annual prevention visits or at every new patient visit) and how often should it be repeated? However, the biggest problem remains that effective selective prevention interventions such as SBI are not widely implemented. Although implementation has worked well in situations where additional grant funds were available, it still is unknown whether physicians will engage in this widely or how to best facilitate implementation. The Veterans Affairs health services system has been the most effective at implementing annual screening, but this system is unique in its structure and hierarchical nature. Implementation of such approaches in private health care organizations is much more complex and difficult. Therefore, more research is needed on low-cost ways to encourage wider adoption of SBI in primary care settings. Additional research should focus on SBI in other medical settings, especially mental health settings and medical specialties particularly affected by heavy drinking, such as gastroenterology (with patients with alcohol-related liver disease, gastritis, and pancreatitis) and otolaryngology (with patients with alcohol-related head and neck cancers).

Because so many hospitalized heavy drinkers have dependence, SBI is much less effective in this group (Saitz et al. 2007) and its effectiveness with patients in EDs or trauma centers also is unknown. Although some early studies showed positive results, subsequent research has yielded as many negative as positive findings (Field et al. 2010). Current efforts to implement SBI in these more acute-care settings therefore are premature, and more research is needed to determine if heavy drinkers encountered in such settings require more intensive services, linkage to ambulatory care services, or both.

People with functional alcohol dependence likely require more than brief counseling, but there is a major gap in research concerning optimal treatment strategies. Currently, few, if any, services are available for this group because they fall between at-risk drinkers and those with severe recurrent alcohol dependence (who are most likely to enter the current specialty treatment system). Pharmacotherapy (e.g., antirelapse medications) combined with medical management offers an attractive possible approach for this group, and evidence suggests that this combination yields comparable results to state-of-the-art counseling (Anton et al. 2006; O’Malley et al. 2003). Such an approach would allow most people with functional dependence to be treated in primary care and mental health care settings, similar to people with mild to moderate depression. More research, especially regarding effectiveness and implementation, is needed on this approach. Although most people with functional alcohol dependence eventually recover without any treatment (Hasin et al. 2007; Moss et al. 2007), their period of illness is associated with less severe but still significant dysfunction, such as absenteeism, attending work or school while sick (i.e., presenteeism), and reduced productivity. Early identification and treatment could reduce or hopefully eliminate these costs to the affected individuals and society.

Gaps in treatment also exist for people with severe recurrent alcohol dependence—the group that most people tend to think of when they think of “alcoholism.” A recent exhaustive report examining the current treatment system concluded that “Most of those who are providing addiction treatment are not medical professionals and are not equipped with the knowledge, skills or credentials necessary to provide the full range of evidence-based services to address addiction effectively,” (p. 3) and that “Addiction treatment facilities and programs are not adequately regulated or held accountable for providing treatment consistent with medical standards and proven treatment practices.” (National Center on Addiction and Substance Abuse at Columbia University 2012, pp. 3–4). The current addiction treatment system first was conceptualized in the middle of the last century, as documented by White (2002), and has changed little since. No other chronic disease is treated with brief stints in a program with limited follow up care. Instead, for other chronic conditions patients are followed closely by physicians and other professionals over long periods of time, with the goal of minimizing symptoms and relapses, treating complications, and maximizing function. In these cases, care is provided indefinitely, often for life. Such a longitudinal-care approach also offers considerable promise in treating people with severe recurrent alcohol dependence. Several studies have found a highly significant positive effect for longitudinal care in people who have one or more medical complications of alcohol dependence (Kristenson et al. 1984; Lieber et al. 2003), including two studies that found significant reduction in 2-year mortality (Willenbring and Olsen 1999; Willenbring et al. 1995). Some findings also indicate that integrating treatment for substance use disorders into that for severe and persistent mental illness may be effective at reducing substance use, although no high-quality randomized controlled trials of this approach have been published (Drake et al. 2006). Pharmacotherapy for AUDs also may be effective in people with severe mental illnesses (Petrakis et al. 2004, 2005, 2006; Salloum et al. 2005). Finally, the ongoing need for recovery support and maintenance should be addressed. Thus, more research is needed on the best long-term management strategies for recurrent alcohol dependence.

## Conclusion

At this time no solid conclusions can be drawn as to whether current approaches to prevention of and treatment for AUDs reduce the disease burden attributable to heavy drinking, although these strategies have shown positive outcomes in the short term. SBI for at-risk drinkers in ambulatory primary care settings has the strongest evidence for efficacy, and some evidence supports its cost-effectiveness and associated reduction in excess morbidity and dysfunction. However, these benefits do not necessarily indicate that health care costs for these patients are reduced. Widespread implementation of SBI for nondependent heavy drinkers outside of the medical context has the potential to have a large public health impact. For heavy drinkers with more severe conditions (i.e., recurrent alcohol dependence), time-limited counseling may improve short-term recovery rates, but its long-term impact is less clear. Moreover, recent research findings have not been widely implemented. Scientifically based, medically anchored treatment approaches may provide a more attractive and cost-effective approach than the current intensive but time-limited treatment. More research is needed on ways to address functional alcohol dependence as well as severe and recurrent alcohol dependence.

## Footnotes

1 The numbers add up to more than 100 percent because respondents could endorse multiple reasons.

2 People with functional alcohol dependence are those who meet the criteria for a medical diagnosis of alcohol dependence but remain functional in society (i.e., in their jobs, families, and social lives).
